# mir-605-3p prevents liver premetastatic niche formation by inhibiting angiogenesis via decreasing exosomal nos3 release in gastric cancer

**DOI:** 10.1186/s12935-024-03359-5

**Published:** 2024-05-27

**Authors:** Yilin Hu, Weijie Zang, Ying Feng, Qinsheng Mao, Junjie Chen, Yizhun Zhu, Wanjiang Xue

**Affiliations:** 1 Department of Gastrointestinal Surgery, Affliated Hospital of Nantong University, Medical School of Nantong University, 20 Xisi Street, Nantong, Jiangsu 226001 China; 2 Research Center of Clinical Medicine, Affiliated Hospital of Nantong University, Nantong, 226001 China; 3 Nantong Key Laboratory of Gastrointestinal Oncology, Nantong, 226001 China; 4 State Key Laboratory of Quality Research in Chinese Medicine, School of Pharmacy, Macau University of Science and Technology, Macau, 999078 China

**Keywords:** miR-605-3p, Exosomes, Premetastatic niche, Angiogenesis, Gastric cancer

## Abstract

**Background:**

Cancer-induced pre-metastatic niches (PMNs) play a decisive role in promoting metastasis by facilitating angiogenesis in distant sites. Evidence accumulates suggesting that microRNAs (miRNAs) exert significant influence on angiogenesis during PMN formation, yet their specific roles and regulatory mechanisms in gastric cancer (GC) remain underexplored.

**Methods:**

miR-605-3p was identified through miRNA-seq and validated by qRT-PCR. Its correlation with the clinicopathological characteristics and prognosis was analyzed in GC. Functional assays were performed to examine angiogenesis both in vitro and in vivo. The related molecular mechanisms were elucidated using RNA-seq, immunofluorescence, transmission electron microscopy, nanoparticle tracking analysis, enzyme-linked immunosorbent assay, luciferase reporter assays and bioinformatics analysis.

**Results:**

miR-605-3p was screened as a candidate miRNA that may regulate angiogenesis in GC. Low expression of miR-605-3p is associated with shorter overall survival and disease-free survival in GC. miR-605-3p-mediated GC-secreted exosomes regulate angiogenesis by regulating exosomal nitric oxide synthase 3 (NOS3) derived from GC cells. Mechanistically, miR-605-3p reduced the secretion of exosomes by inhibiting vesicle-associated membrane protein 3 (VAMP3) expression and affects the transport of multivesicular bodies to the GC cell membrane. At the same time, miR-605-3p reduces NOS3 levels in exosomes by inhibiting the expression of intracellular NOS3. Upon uptake of GC cell-derived exosomal NOS3, human umbilical vein endothelial cells exhibited increased nitric oxide levels, which induced angiogenesis, established liver PMN and ultimately promoted the occurrence of liver metastasis. Furthermore, a high level of plasma exosomal NOS3 was clinically associated with metastasis in GC patients.

**Conclusions:**

miR-605-3p may play a pivotal role in regulating VAMP3-mediated secretion of exosomal NOS3, thereby affecting the formation of GC PMN and thus inhibiting GC metastasis.

**Supplementary Information:**

The online version contains supplementary material available at 10.1186/s12935-024-03359-5.

## Introduction

Gastric cancer (GC) is a common malignant tumor of the digestive tract with high rates of distant metastasis at diagnosis [1]. Limited treatment options and poor prognoses are observed [2]. The formation of premetastatic niches (PMNs) in distant organs plays a crucial role in tumor metastasis [3]. Angiogenesis facilitates PMN formation and tumor cell migration [4]. Therefore, studying the angiogenesis mechanism of GC, developing effective treatment strategies, and improving the prognosis of patients with distant GC are essential. Studies have reported that tumour cells release VEGFs [5], NFAT2 [6], ANG2, MMP-1, MMP-2, MMP-3, MMP-10, EGFRs, COX-2, extracellular vesicles (EVs) and other mediators to transmit information to target organ cells [7, 8], thereby altering the angiogenesis of the target organ and promoting PMN formation.

Exosomes are a type of EV approximately 30–150 nm in diameter and are the main source of intercellular communication. Because they can achieve long-distance, cross-organ targeted transport, EVs are considered one of the key links in the formation of PMN induced by tumour cells [9]. The generation of exosomes starts with the invagination of the plasma membrane. In the process of maturation from early endosomes to multivesicular bodies (MVBs), endosomes produce intraluminal vesicles (ILVs) through inward budding, forming the precursors of exosomes [10]. In the process of exosome secretion outside of the cell, Ras-related proteins in brain (Rab) and soluble NSF-attachment protein receptor (SNARE) families play important auxiliary roles. For example, VAMP3, VAMP7, and YKT6 affect exosome secretion by regulating the exocytosis of tumor cells [11, 12]. In liver cancer, the complex of SNAP23 and VAMP3 promotes the secretion of exosomes in the tumor microenvironment (TME) [13]. However, the upstream regulatory mechanisms of exosomes are not well understood.

MicroRNAs (miRNAs) are a type of endogenous noncoding RNA with a length of approximately 22 nt. They can negatively regulate the expression of target genes to affect multiple pathophysiological processes, including the occurrence, development, and metastasis of GC [14]. miRNAs shown to be directly transcriptionally activated by p53 can play an important role in influencing angiogenesis [15]. miR-34a was shown to ameliorate VEGF-mediated retinal angiogenesis by targeting Notch1 [16]. Adipose-derived stem cell (ASC)-secreted EVs can carry miR-486-5p to promote wound healing by stimulating angiogenesis [17]. miR-145 regulates pathological retinal angiogenesis by suppressing Tropomodulin3 (TMOD3) [18]. Exosomal miR-205 derived from ovarian cancer cells promotes metastasis by inducing angiogenesis via the PTEN-AKT pathway [19]. Endothelial miR-26a regulates VEGF-Nogo-B receptor-mediated angiogenesis by modulating nitric oxide synthase 3 (NOS3) activity [20]. However, whether these miRNAs affect PMN formation via angiogenesis in GC has not yet been elucidated.

In this study, we found that exosomal miR-605-3p does not directly affect PMN formation in GC. Instead, it modulates VAMP3 and NOS3 expression in GC cells, impacting exosome secretion and exosomal NOS3 expression and transport. These changes influence distant organ angiogenesis and promote PMN formation, providing new insights for GC metastasis treatment.

## Materials and methods

### Patients and sample collection

This study was approved by the ethics committee of the Affiliated Hospital of Nantong University. Included patients were assigned to two cohorts. Cohort 1 comprised 108 GC patients undergoing D2 radical surgery between March and December 2010 at the Affiliated Hospital of Nantong University. For each patient, a GC specimen and a paired healthy specimen were collected and analysed. The patients were followed until March 2016, with an average follow-up period of 40 ± 23 months. GC patients without metastases to other organs before surgery who underwent surgery but did not receive adjuvant therapy, radiotherapy, or immunotherapy were included in the cohort. Among the patients in this cohort, 8 experienced tumour recurrence within one year, and 54 did not present tumour relapse within 5 years. Cohort 2 comprised 80 healthy individuals, 80 GC patients without distant metastasis detected, and 20 GC patients with distant metastasis (samples collected between 2018 and 2019). Plasma samples were collected from these patients both before and after surgery. Enhanced computed tomography (CT) of the abdomen was performed to determine whether these patients harboured detectable distant metastases.

### Cell lines and culture

The SGC-7901, BGC-823, HGC-27, MKN-45, and AGS cell lines, a normal gastric mucosal epithelial cell line (GES-1), the HEK293T cell line and human umbilical vein endothelial cells (HUVECs) were ordered from GeneChem (Shanghai, China). The SGC-7901, BGC-823, MKN-45, HGC-27, AGS, and GES-1 cell lines were cultured in RPMI 1640 medium (Gibco, Gaithersburg, MD, USA), while HUVECs were cultured in endothelial cell medium (ScienCell Research Laboratories, Shanghai, China) and HEK293T cells were cultured in DMEM (Gibco) supplemented with 10% foetal bovine plasma (FBS, Clark, Shanghai, China) and 100 U/mL penicillin (Thermo Fisher, Waltham, USA). Conditioned medium (CM) collected from RPMI 1640-cultured AGS and SGC-7901 cells without FBS for 2 days was stored at -80 °C until further use. HUVECs were incubated in CM supplemented with 10% exosome-depleted FBS.

### Cell transfection

Expression vectors encoding NOS3 or VAMP3 and empty vectors were obtained from GeneChem (Shanghai, China). The miR-605-3p mimic/ miR-605-3p mimic negative control (NC)/ miR-605-3p inhibitor/ miR-605-3p inhibitor negative control (miR-NC) were purchased from GenePharma (Suzhou, China). siRNA sequences corresponding to si-NOS3 and si-VAMP3 were synthesized by GenePharma and are listed in Table S1. Plasmids or oligonucleotides were transfected into the designated cells using JetPRIME (Polyplus) or GP-transfect-Mate (GenePharma) reagents. For double transfection, the plasmids or oligonucleotides were introduced sequentially into the target cells at 24-hour intervals.

### RNA extraction and quantitative real-time polymerase chain reaction (qRT-PCR)

RNA extraction and qRT-PCR were carried out as described previously [21]. The primer sequences are listed in Table S2 [22–27].

### Western blot (WB)

Protein extraction and WB assays were performed as previously described [28]. Primary antibodies targeting the following proteins were used in our WB analysis: Lamp2 (Proteintech, Wuhan, China, 27823-1-AP, 1:2000), EEA1 (Proteintech, 28347-1-AP, 1:2000), LC3B (Proteintech, 18725-1-AP, 1:2000), CD63 (Abcam, Burlingame, CA, USA, ab134045, 1:2000), GAPDH (Proteintech, 60004-1-Ig, 1:2000), NOS3 (Proteintech, 27120-1-AP, 1:2000), VAMP3 (Proteintech, 10702-1-AP, 1:2000), TSG101 (Proteintech, 28283-1-AP, 1:2000), Calnexin (Proteintech, 10427-2-AP, 1:2000).

### RNA sequencing analysis and bioinformatics analyses

Seq1: To further explore the potential miRNAs regulating angiogenesis, we conducted miRNA sequencing on AGS and SGC-7901 cells using the Illumina sequencing platform provided by Genedenovo Biotechnology Co., Ltd. (Guangzhou, China). For annotation and analysis, the miRBase database (https://mirbase.org/) served as the reference genome for human miRNA sequences. Seq2: To elucidate the role of miR-605-3p in angiogenesis further, we transfected SGC-7901 cells with either the miR-605-3p mimic or a negative control. We then performed RNA sequencing to compare the transcriptomic profiles of cells treated with the miR-605-3p mimic versus the negative control, using three biological replicates for each condition. Seq3: In an effort to uncover the mechanisms through which conditioned medium from AGS cells, treated with either a miR-605-3p inhibitor or a negative control, distinctly affects angiogenesis in HUVECs, we conducted RNA sequencing. HUVECs were incubated with an equivalent number of exosomes derived from the conditioned medium of both the AGS/miR-605-3p inhibitor and AGS/miR-NC groups, with RNA-seq analysis performed on three biological replicates per group. Sequencing for Seq2 and Seq3 was carried out by Genewiz (Soochow, China). Gene set enrichment analysis (GSEA) generated two ordered lists—one including all related genes based on correlation with miR-605 and the other comprising upregulated genes determined in transcriptome sequencing. “h.all.v6.2.entrez.gmt” was selected as the reference gene set [29]. Gene ontology (GO) analyses were performed using high-throughput sequencing data based on biological processes, molecular functions and cellular components. Gene set permutations were performed 1000 times for each analysis.

### Isolation, treatment and characterization of exosomes

Exosomes from CM and plasma were obtained as previously described [30], fixed with 2% paraformaldehyde, and placed on a 200 mesh Formvar-coated grid. This grid was stained with 2% phosphotungstic acid for 2 min, after which the exosomes could be observed. For fluorescent staining, PKH67 membrane dye (Sigma) was used to label exosomal membranes. Labelled exosomes were cleared by supercentrifugation and incubated with HUVECs for 6 h before the cultures were observed, as previously described [31]. The size and number of exosomes were detected by nanoparticle tracking analysis (NTA) using a NanoSight NS500 instrument (Malvern Panalytica, UK). A 488 nm laser camera was equipped to track Brownian motion and particle size for 60 s by producing a video. The videos were analysed using the NTA2.3 software. The concentration of exosomes was tested by BCA protein Assay Kit (Termo Scientifc, Rockford, lot. 23,235, USA). The surface marker proteins of exosomes were detected using WB.

### Transmission electron microscopy (TEM)

First, 20 µL of freshly extracted exosomes was dried in a copper grid for 15 min. Then, the samples were fixed in 2.5% glutaraldehyde for 1 h and stained with 3% phosphotungstic acid for 1 min before immediately observation under a TEM (JEOL Ltd, Tokyo, Japan, JEM-1230). Cells treated with miR-605-3p mimic or inhibitor were washed with PBS and fixed with glutaraldehyde for 2 h at 4 °C. Then, the cells were incubated with 1% agar solution at 40 °C before observation under a transmission electron microscope. The MVBs and ILVs identified by TEM were described previously [32, 33].

### Immunohistochemistry (IHC)

IHC was performed according to a previous protocol [34]. Tumour sections of human GC tissue and nude mouse xenografts and liver samples were analysed by IHC using the EnVision™ System (Dako, Carpinteria, CA). Photographed using Zeiss Axio Vert5 (Jena, Germany). Paraffin-embedded slides were incubated with antibodies targeting CD31 (Abcam, ab155607, 1:100), NOS3 (Abcam, ab252439, 1:100) and VAMP3 (Proteintech, 10702-1-AP, 1:200). The IHC scores were calculated using the average proportion of positively stained cells over five random fields. The IHC results were evaluated by two independent pathologists, and scores were recorded based on the staining intensity (3, strongly positive; 2, moderately positive; 1, weakly positive; and 0, negative) and percentage of positive cells (4, > 75%; 3, > 50-75%; 2, > 25-50%; 1, 5-25%; and 0, < 5%). The final score was calculated by multiplying the intensity and percentage scores, with high expression defined as a score ≥ 3 and low expression defined as a score ≤ 2 [35, 36]. Microvascular density (MVD) was assessed according to the Weider determination method [37]. After observing the vascular distribution in the whole section under a 100x microscope, areas with high MVD were selected and counted in 5 fields under a 400x microscope. The average value was recorded as the MVD. Vessels with a brown–yellow endothelium were counted as microvessels as long as they were separated from peripheral blood vessels, tumour cells, and other connective tissue.

### Tube formation assays

HUVECs were treated with exosomes (10 µg exosomes or exosomes extracted from 1 × 10^7^ GC cells in 100 µl PBS). For the tube formation assay, treated HUVECs (1 × 10^4^/well) were seeded into a 96-well plate (NEST, Wuxi, China) according to the manufacturer’s recommendations. Tube-like structures were viewed under an inverted microscope 2 h after seeding. Finally, HUVECs were fluorescently stained with Calcein AM (Beyotime, Shanghai, China). The tube formation ability was quantified using ImageJ (version 4.1) and Angiogenesis Analyzer plugin.

### Enzyme-linked immunosorbent assay (ELISA)

After the corresponding treatment, GC cells were starved for 24 h, CM was collected and centrifuged (1500 rpm, 5 min, 4℃). The whole blood was centrifuged (1000 g, 10 min, 4℃) and plasma was collected. ELISA kits were used to determine exosomal NOS3 (Jianglaibio, Shanghai, China, JL18188) and NO (Jianglaibio, JL15362) secretion according to manufacturer’s instructions.

### Immunofluorescence

HUVECs seeded onto a cell climbing tablet in a 24-well plate were fixed, permeabilized, blocked, and incubated overnight at 4 °C with primary antibodies targeting the following proteins: Tubulin (Proteintech, 11224-1-AP, 1:100) and CD63 (Abcam, ab217345, 1:100). Then, the cells were incubated with Alexa Fluor 594-conjugated goat anti-rabbit IgG (ABclonal, Wuhan, China) and Alexa Fluor 488-conjugated goat anti-mouse IgG (Abclonal, Wuhan, China) before they counterstaining with 4′,6-diamidino-2-phenylindole (DAPI, Invitrogen, California, USA). Photographed using Zeiss Axio Vert5 (Jena, Germany).

### Luciferase reporter assay

For conducting luciferase assays, the 3′-untranslated regions (3′-UTRs) of both VAMP3 and NOS3, along with their respective 3′-UTRs harboring a mutated predicted binding site, were inserted into the pmirGLO luciferase reporter plasmid (GeneChem, Shanghai, China). 3 × 10^4^ HEK293T or SGC-7901 cells per well were seeded in 24-well plates and allowed to culture for a period of 24 h. Subsequently, either VAMP3-WT or VAMP3-MUT, and separately NOS3-WT or NOS3-MUT, were co-transfected alongside either the miR-605-3p mimic or the miR-605-3p negative control (NC). 48 h post-transfection, the Dual Luciferase Assay Kit (Vazyme, Nanjing, China) was utilized to measure the luciferase reporter activities following the manufacturer’s instructions.

### Nude mice models

For exosome absorbing assay, 50 µg exosomes labelled by PKH67 from GC cells were injected into the tail vein of 4-week-old nude mice every two days for 14 days. For PMN nude mice model, 50 µg exosomes from GC cells or exosomes from 4 × 10^7^ GC cells were treated with the same groups, as follows: AGS/miR-NC, AGS/miR-605-3p inhibitor, AGS/miR-605-3p inhibitor + si-VAMP3, AGS/miR-605-3p inhibitor + si-NOS3. Then they were injected into the tail vein of 4-week-old nude mice every two days for 14 days (*n* = 5 per cell type). Then, AGS cells were injected into the spleen capsule of remaining mice. After 4 weeks, the animals were sacrificed, livers were removed for IHC or H&E staining. These studies were approved by the Laboratory Animals Center of Nantong University. All nude mice were purchased from the Animal Center of the Medical College of Nantong University.

### Statistical analysis

Statistical analyses were performed using SPSS (version 23.0, IBM, Chicago, IL, USA) and GraphPad Prism (version 8.0, GraphPad Software, San Diego, USA). The quantitative values of all experiments are expressed as the mean ± SD. Differences between two sample groups were analysed by an independent samples t-test, and differences among more than two sample groups were analysed by one-way analysis of variance (ANOVA). Pearson’s correlation coefficient was employed to measure the linear correlations between miR-605-3p and CD31, MVD and VAMP3, exosomal NOS3 in plasma and NOS3 in tissues. *P* < 0.05 was considered statistically significant.

## Results

### Mir-605-3p is associated with angiogenesis in GC

**Fig. 1:**
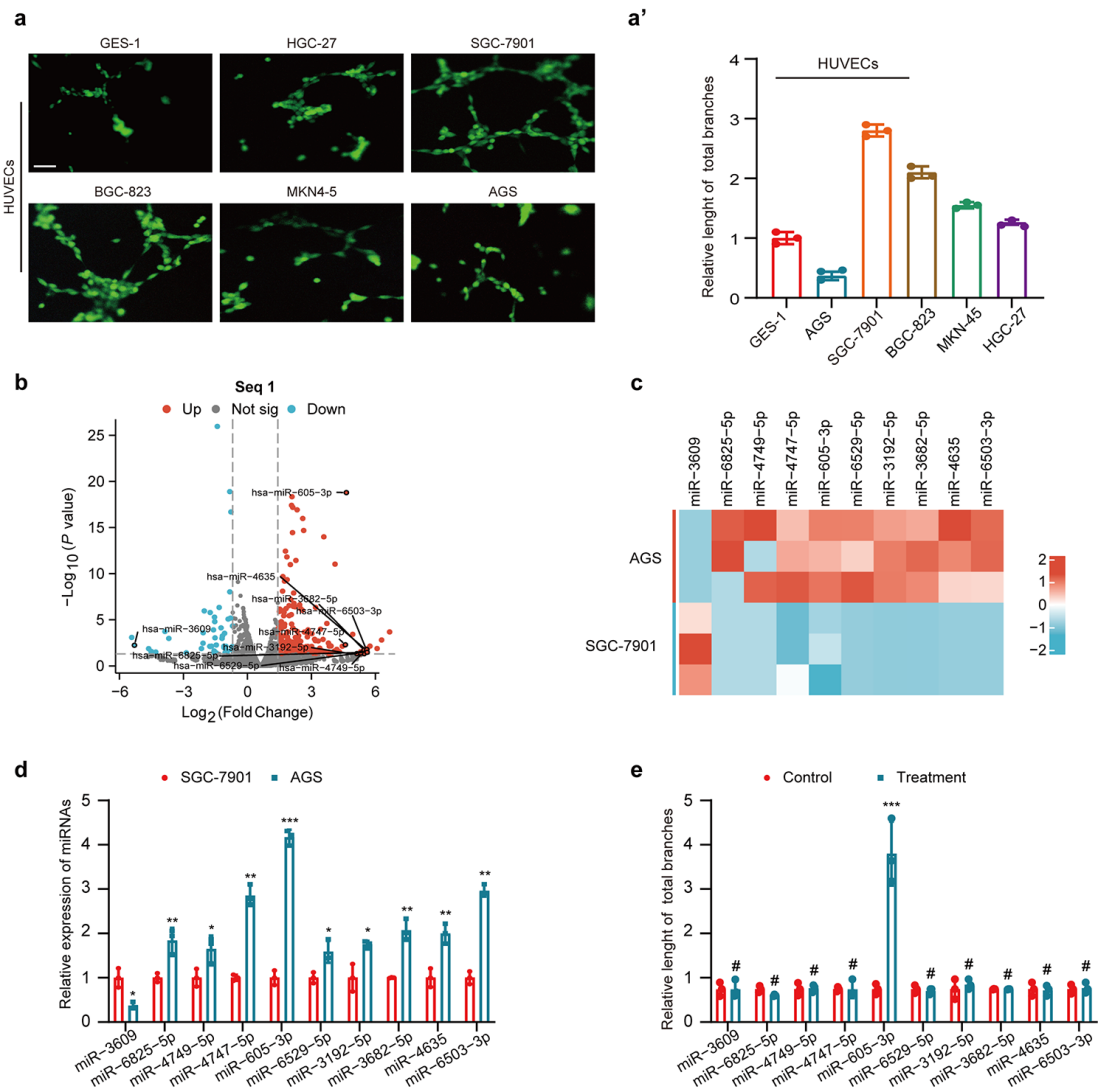
miR-605-3p is associated with angiogenesis in GC. **(a)** Effect of CM derived from different GC cells on tube formation ability (scale bar, 100 μm). **(b)** Volcano plot of miRNA-seq (Seq1) showed miRNAs with significant up- and down-regulation of expression. **(c)** Heat maps showed the top 10 exist miRNAs that were most differentially expressed in Seq1(AGS vs. SGC-7901). **(d)** qRT-PCR analysis of miRNA expression in SGC-7901 and AGS. **(e)** Effects of conditioned medium (CM) derived from AGS cells treated with miR-NC, miR-3609 mimic, miR-6825-5p inhibitor, miR-4749-5p inhibitor, miR-4747-5p inhibitor, miR-605-3p inhibitor, miR-6529-5p inhibitor, miR-3192-5p inhibitor, miR-3682-5p inhibitor, miR-4635 inhibitor, miR-6503-3p inhibitor on the tube formation ability of HUVECs. *P < 0.05, **P < 0.01 and ***P < 0.001

To identify miRNAs involved in regulating angiogenesis in GC, we used SGC-7901 and AGS cell lines. SGC-7901 exhibited strong angiogenic stimulation in HUVECs compared to the weaker stimulation by AGS, among five gastric cancer cell lines (Fig. 1a). We conducted miRNA sequencing (miRNA-seq, Seq1) on these cell lines to identify differentially expressed miRNAs associated with angiogenesis regulation in GC (Fig. 1b). Among the top ten differentially expressed miRNAs (Fig. 1c), validated by qRT-PCR (Fig. 1d), only miR-605-3p overexpression affected GC angiogenesis (Fig. 1e and Supplementary Fig. 1a). These findings indicate that miR-605-3p may play a significant role as a key miRNA in regulating angiogenesis in gastric cancer.

### Low expression of mir-605-3p in GC patients is associated with MVD and predicts poor prognoses

**Fig. 2:**
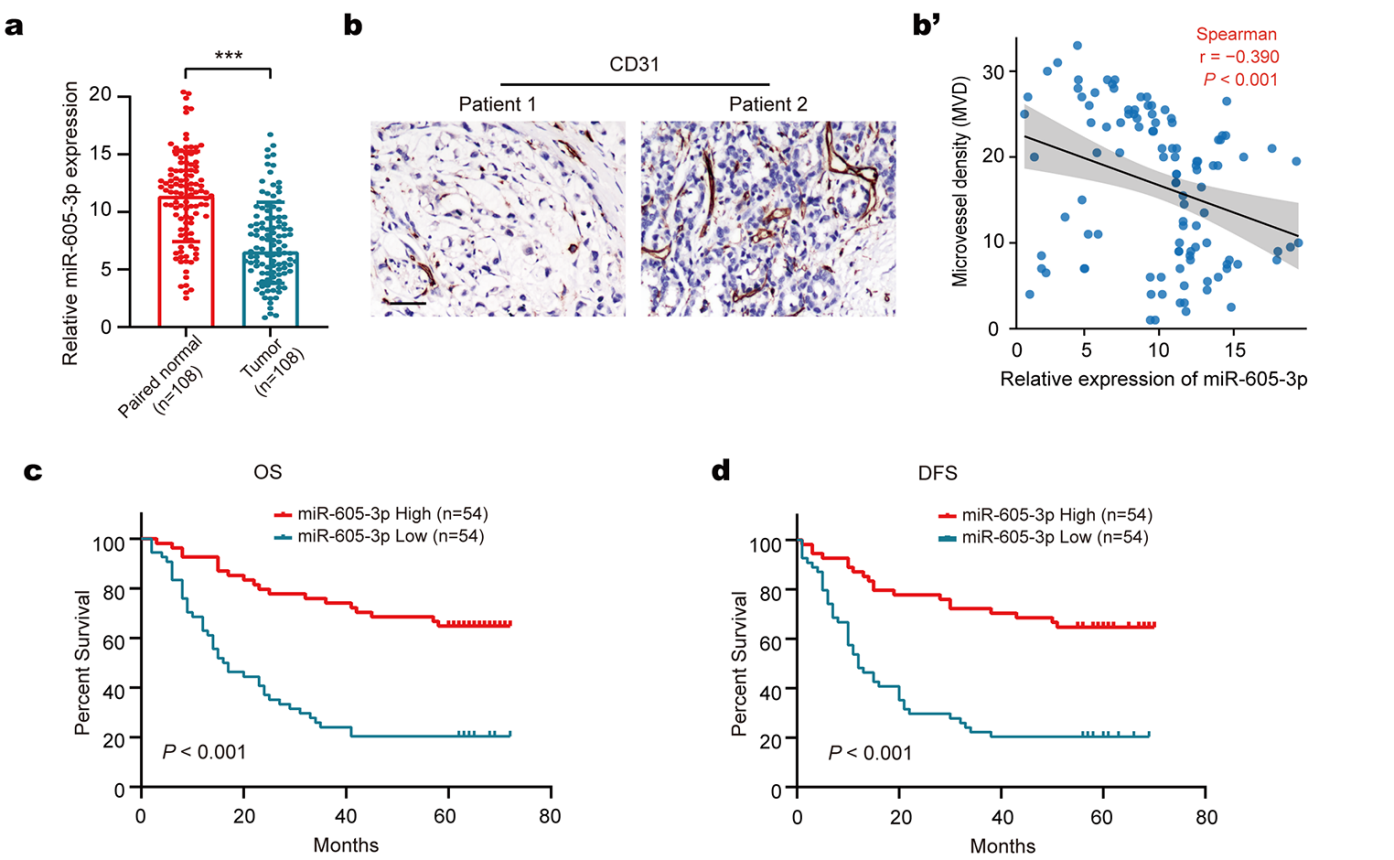
Low expression of miR-605-3p in GC patients is associated with MVD and predicts poor prognoses. **a.** qRT-PCR analysis of miR-605-3p expression in GC tissues and adjacent normal tissues from 108 GC patients. **b.** CD31 determined by IHC in GC tissues (scale bar, 50 µm). **b’**. The expression of miR-605-3p correlated negatively with MVD. **c, d** Kaplan–Meier analysis of OS (**c**) and DFS (**d**) in GC patients stratified by miR-605-3p expression. ***P < 0.001

To explore the clinical relevance of miR-605-3p in GC, we analyzed 108 pairs of GC tissue samples (Fig. 2a). MiR-605-3p levels were downregulated in GC tissue, and its expression was associated with N stage, tumor size, T stage, TNM stage, and MVD (Table 1). Multivariate analysis revealed that low miR-605-3p expression was significantly associated with N stage (*P* = 0.001; HR = 0.066; 95% CI: 0.015–0.279) and MVD (*P* = 0.013; HR = 0.237; 95% CI: 0.077–0.735). The negative correlation between miR-605-3p expression and MVD was confirmed (Fig. 2b). Kaplan–Meier survival analysis showed that low miR-605-3p expression correlated with shorter overall survival (OS) and disease-free survival (DFS) times (Fig. 2c, d). Multivariate analysis confirmed miR-605-3p’s independent prognostic significance for shorter OS and DFS in GC patients (Table 2). In summary, our findings indicate that miR-605-3p is associated with MVD and predicts poor outcomes in GC patients.

**Table 1:** Relationship between GC patient characteristics and miR-605-3p level (*P < 0.05)

Clinicalpathological fetures	miR-605-3p level		Total	*P* value
	High(*n* = 54)	Low(*n* = 54)		
**Gender**				0.296
Male	40	35	75	
Female	14	19	33	
**Age(years)**				0.620
≤ 60	9	11	20	
> 60	45	43	88	
**Tumor diameter(cm)**				0.054
≤ 4	34	24	58	
> 4	20	30	50	
**Tumor location**				0.433
Up/ Middle	20	24	44	
Down	34	30	64	
**Tumor differentiation**				0.563
High/ Moderate	27	30	57	
Low	27	24	51	
**TNM stage**				0.002*
I/II	36	20	56	
III	18	34	52	
**T stage**				0.003*
1/2	28	13	41	
3/4	26	41	67	
**N stage**				0.001*
0/1	43	26	69	
2/3	11	28	39	
**MVD**				<0.001*
High	18	38	56	
Low	36	16	52	

**Table 2:** Univariate and multivariable analyses of OS and DFS in GC patients (*P < 0.05)

Variable	OS			DFS		
	Univariate analysis	Multivariable analysis		Univariate analysis	Multivariable analysis	
	*P*>|z|	*P*>|z|	HR (95%CI)	*P*>|z|	*P*>|z|	HR (95%CI)
**miR-605-3p** **expression**						
High (*n* = 54) vs. Low (*n* = 54)	**< 0.001**	0.009*	0.456(0.253–0.823)	**< 0.001**	0.014*	0.477(0.264–0.861)
**Gender**						
Male (*n* = 75) vs. female (*n* = 33)	0.105			0.117		
**Age (years)**						
≤ 60 (*n* = 20) vs. >60 (*n* = 88)	0.512			0.521		
**Tumor differentiation**						
High/ Moderate (*n* = 57) vs. Low (*n* = 51)	0.108			0.110		
**Tumor diameter (cm)**						
≤ 4 (*n* = 58) vs. >4 (*n* = 50)	0.027*			0.032*		
**TNM stage**						
1/2(*n* = 41) vs. 3/4 (*n* = 67)	< 0.001*	**——**		< 0.001*	**——**	
**N stage**						
0/1 (*n* = 69) vs. 2/3 (*n* = 39)	< 0.001*	0.004*	2.193(1.277–3.767)	< 0.001*	0.006*	2.126(1.237–3.654)
**Tumor location**						
Up/Middle (*n* = 44) vs. Down (*n* = 64)	0.516			0.511		
**MVD**						
High (*n* = 56) vs. Low (*n* = 52)	< 0.001*	0.001*	2.950(1.534–5.673)	< 0.001*	0.001*	2.916(1.520–5.595)

### Mir-605-3p suppresses GC angiogenesis by inhibiting the release of exosomes

**Fig. 3:**
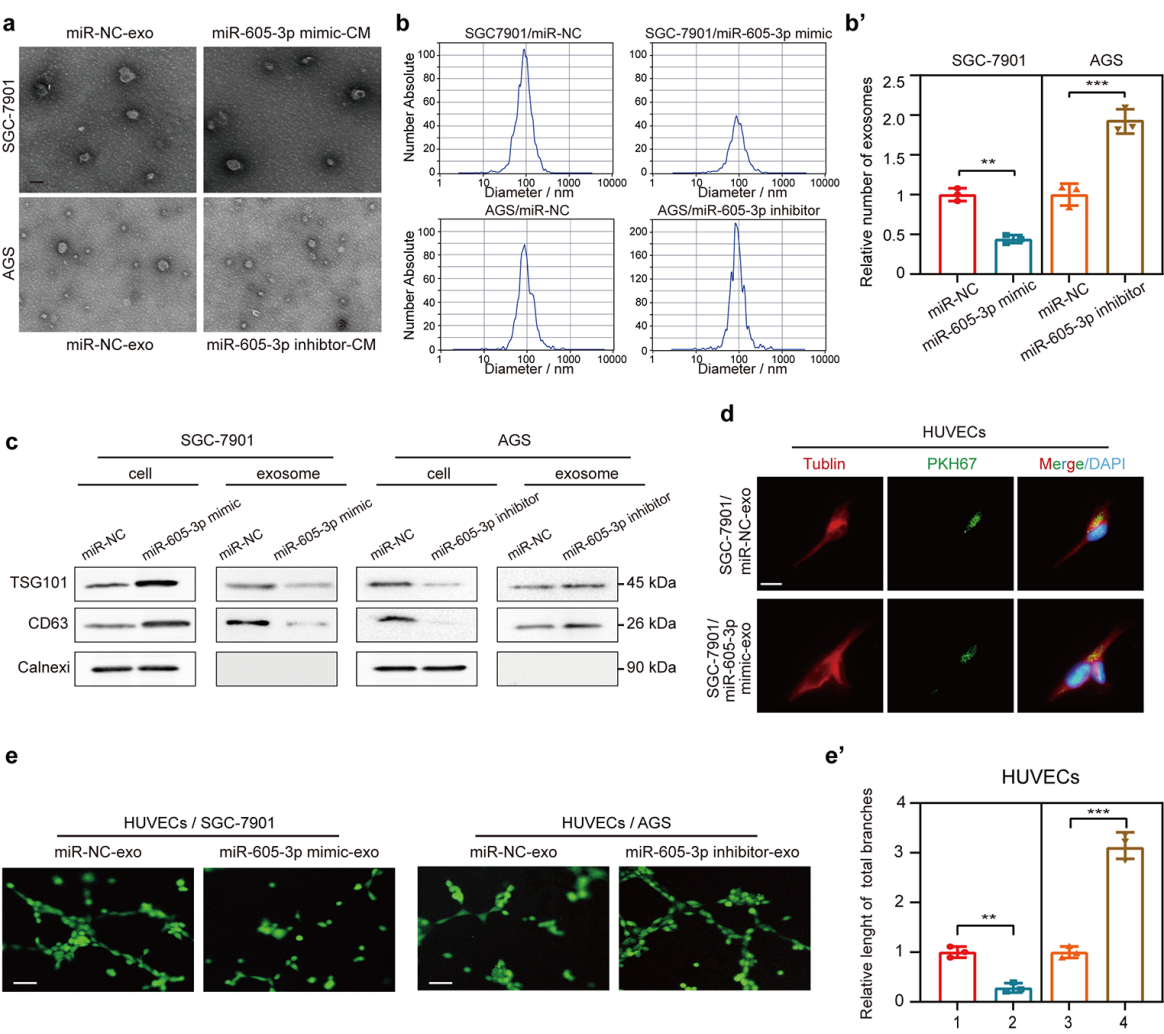
miR-605-3p suppresses GC angiogenesis by inhibiting the release of exosomes. **(a)** Electron microscopy scans of exosomes derived from SGC-7901/miR-NC, SGC-7901/miR-605-3p mimic, AGS/miR-NC, and AGS/miR-605-3p inhibitor cells (scale bar, 100 nm). **(b)** Characterization and quantitation of vesicles derived from the same number of SGC-7901/miR-NC, SGC-7901/miR-605-3p mimic, AGS/miR-NC, and AGS/miR-605-3p inhibitor cells as assessed by NTA. **(c)** WB was performed to evaluate the expression of the exosomal markers CD63 and TSG101 and the marker calnexin of lysates from the same number of GC cells. **(d)** Fluorescence of HUVECs incubated for 48 h with PKH67-labelled exosomes derived from SGC-7901/miR-NC and SGC-7901/miR-605-3p mimic cells (scale bar, 25 μm). **(e)** Effect of exosomes derived from the same number of SGC-7901/miR-NC, SGC-7901/miR-605-3p mimic, AGS/miR-NC, and AGS/miR-605-3p inhibitor cells on tube formation ability (scale bar, 100 μm). **P < 0.01, ***P < 0.001

To investigate the mechanism of miR-605-3p inhibition on angiogenesis, we examined the expression of several angiogenesis regulatory molecules in GC cells [7]. However, no significant changes were observed in the miR-605-3p overexpression group compared to the control group (Supplementary Fig. 1b and 1c). Since exosomes have been shown to regulate angiogenesis [38], we examined the effect of exosomes derived from miR-605-3p-modulated GC cells on HUVEC behavior. Exosomes were extracted from CM of SGC-7901/miR-NC, SGC-7901/miR-605-3p, AGS/miR-NC and AGS/miR-605-3p inhibitor groups with the same number of cells. These exosomes were membrane-encapsulated particles of size 50–150 nm, as confirmed by TEM and NTA (Fig. 3a and b). NTA and WB analysis showed that the number of exosomes in the SGC-7901/miR-605-3p group was significantly reduced compared with the control group, while the number of exosomes in the AGS/miR-605-3p inhibitor group was increased under the same cell number (Fig. 3b and c). However, the pattern was reversed in the cell lysate (Fig. 3c). These results suggest that miR-605-3p regulates exosome release in GC cells.

Subsequently, HUVECs were cocultured with PKH67-labeled exosomes, demonstrating efficient uptake by HUVECs (Fig. 3d). Further analysis showed that in the same number of GC cells, exosomes from miR-605-3p mimic cells reduced the tube formation ability of HUVEC and weakened the migration and invasion ability of HUVEC, while exosomes from miR-605-3p inhibitor cells showed the opposite effect (Fig. 3e and Supplementary Fig. 1d-e). These findings demonstrated that miR-605-3p suppressed angiogenesis by inhibiting the secretion of exosomes from GC cells.

### Mir-605-3p inhibited the release of exosomes by regulating VAMP3 expression

**Fig. 4:**
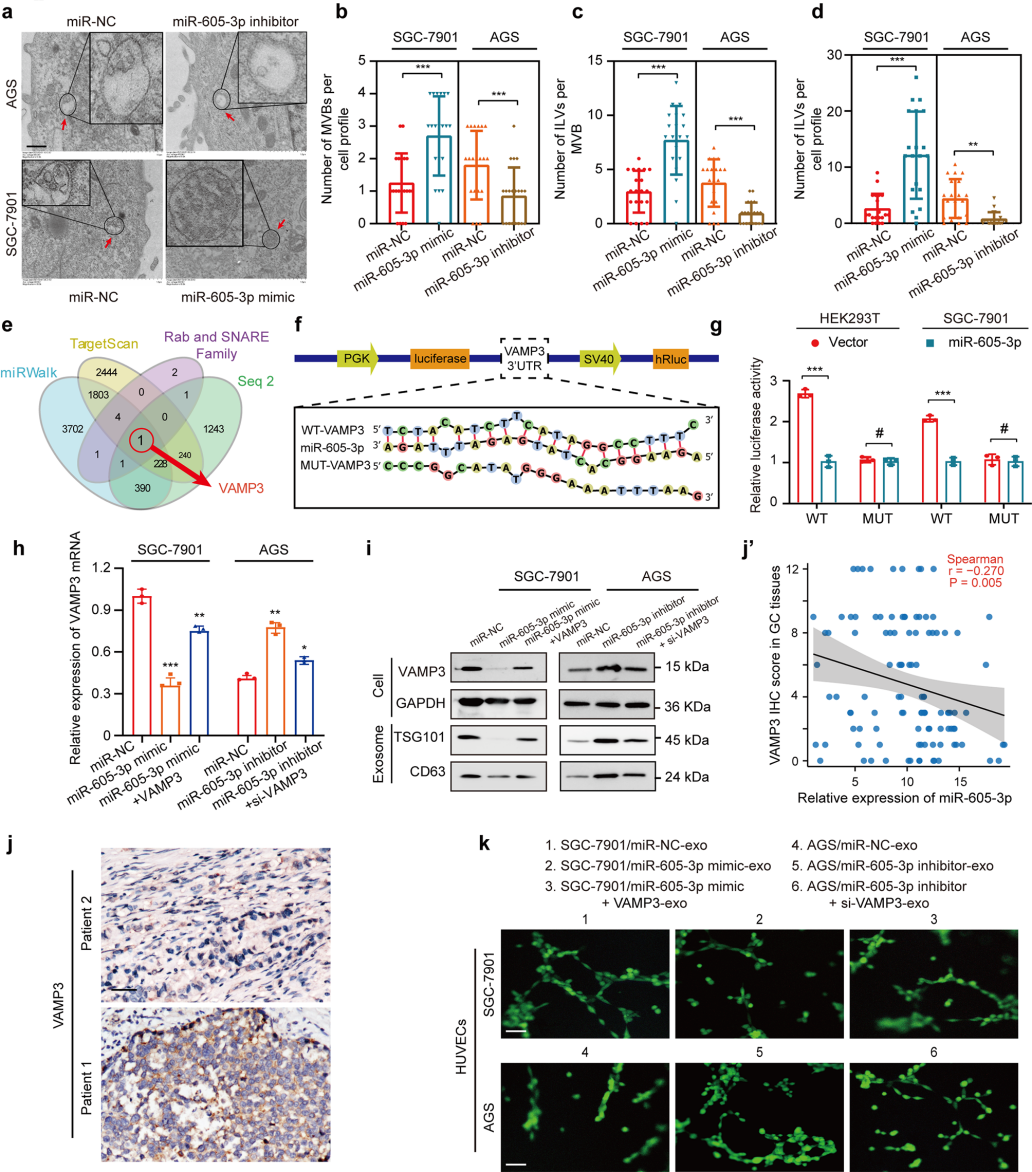
miR-605-3p inhibited the release of exosomes by regulating the expression of VAMP3 in GC cells. **a.** Electron microscopy images of MVBs from SGC-7901/miR-NC, SGC-7901/miR-605-3p mimic, AGS/miR-NC, and AGS/miR-605-3p inhibitor cells (scale bar, 5 µm). **b**. Number of MVBs per cell profile. **c**. Number of ILVs per MVB. **d**. Number of ILVs per cell profile. Only MVBs containing typical ILVs were counted. **e**. The Venn diagram displays the intersection results of miRNA target gene predictions based on online databases (TargetScan and miRWalk) and differential genes (P < 0.05) from transcriptome sequencing (Seq2) of GC-7901/miR-605-3p mimic vs. SGC-7901/NC. **f**. Predicted target sequence of miR-605-3p in the wild-type (WT) 3′-UTR of VAMP3 mRNA and sequences containing altered nucleotides in the predicted binding site for miR-605-3p. **g.** Luciferase reporter assay of HEK-293T and SGC-7901 cells transfected with miR-605-3p mimic and a reporter vector containing the WT or mutated (Mut) VAMP3 3′-UTR. **h**. qRT-PCR assessed VAMP3 mRNA levels in the SGC-7901/miR-NC, SGC-7901/miR-605-3p mimic, SGC-7901/miR-605-3p mimic + VAMP3, AGS/miR-NC, AGS/miR-605-3p inhibitor, and AGS/miR-605-3p inhibitor + si-VAMP3 cells. **i.** WB assessed VAMP3 protein level in GC cells or exosomes from same number of GC cells in the same treatment groups as mentioned above. **j.** VAMP3 determined by IHC in GC tissues (scale bar, 50 µm). **j’.** Expression of miR-605-3p correlated negatively with that of VAMP3 in 108 cases of GC. **k**. Effect of exosomes derived from the same number of SGC-7901/miR-NC, SGC-7901/miR-605-3p mimic, SGC-7901/miR-605-3p mimic + VAMP3, AGS/miR-NC, AGS/miR-605-3p inhibitor, and AGS/miR-605-3p inhibitor + si-VAMP3 cells (scale bar, 100 μm) on the tube formation ability of HUVECs. *P < 0.05, **P < 0.01, ***P < 0.001, ^#^*P* no significance

To investigate the regulation of exosome release by miR-605-3p in GC cells, we examined MVB morphology. TEM analysis revealed decreased MVBs and ILVs in AGS/miR-605-3p inhibitor cells and increased MVBs and ILVs in SGC-7901/miR-605-3p mimic cells (Fig. 4a-d). No morphological differences were observed. Early endosome, lysosome, and autophagosome markers showed no significant changes (Supplementary Fig. 2a-c).

Exosome release from MVBs to the extracellular space relies on Rab (Rab5a, Rab5b, Rab7a, Rab11a, Rab27a, Rab27b, and Rab35) and SNARE (VAMP3, VAMP7, and YKT6) protein families [39]. miRNA usually exert their functions by competitive binding to the downstream mRNA [20]. Based on the miRNA target gene prediction online databases (TargetScan: https://www.targetscan.org/ and miRWalk: http://mirwalk.umm.uni-heidelberg.de/) and differential genes (*P* < 0.05) from transcriptome sequencing (Seq2) of GC-7901/miR-605-3p mimic vs. SGC-7901/NC. we predicted the downstream target gene VAMP3 of miR-605-3p (Fig. 4e and Supplementary Fig. 2d). A predicted binding site for miR-605-3p was identified in the VAMP3 3’-UTR (Fig. 4f). Luciferase reporter assays confirmed this interaction (Fig. 4g). Furthermore, qPCR and Western blot assays showed that miR-605-3p overexpression down-regulated VAMP3 expression in SGC-7901 cells, while miR-605-3p inhibitor up-regulated VAMP3 expression in AGS cells (Fig. 4h and i). Meanwhile, NTA and Western blot experiments demonstrated that, under the same cell number conditions, overexpression of VAMP3 partially restored the reduced exosome secretion caused by miR-605-3p overexpression in SGC-7901 cells, whereas VAMP3 knockdown partially reversed the increased exosome secretion resulting from miR-605-3p underexpression in AGS cells (Fig. 4i, Supplementary Fig. 3a). In addition, IHC results showed that VAMP3 expression was negatively correlated with miR-605-3p in GC tissues (Fig. 4j).

Tube formation assays using exosomes from different treatment groups with the same number of GC cells showed that exosomes from SGC-7901/miR-605-3p mimic/ VAMP3 cells significantly enhanced HUVEC tube formation ability compared to exosomes from miR-605-3p mimic cells. Conversely, exosomes from AGS/miR-605-3p inhibitor/si-VAMP3 cells exhibited significantly weaker tube formation ability compared to exosomes from AGS/miR-605-3p inhibitor cells (Fig. 4k, Supplementary Fig. 3b). In summary, miR-605-3p inhibited the secretion of exosomes from GC cells by regulating VAMP3 expression.

### Mir-605-3p suppresses angiogenesis by targeting NOS3 and regulating exosomal NOS3 derived from GC cells

**Fig. 5:**
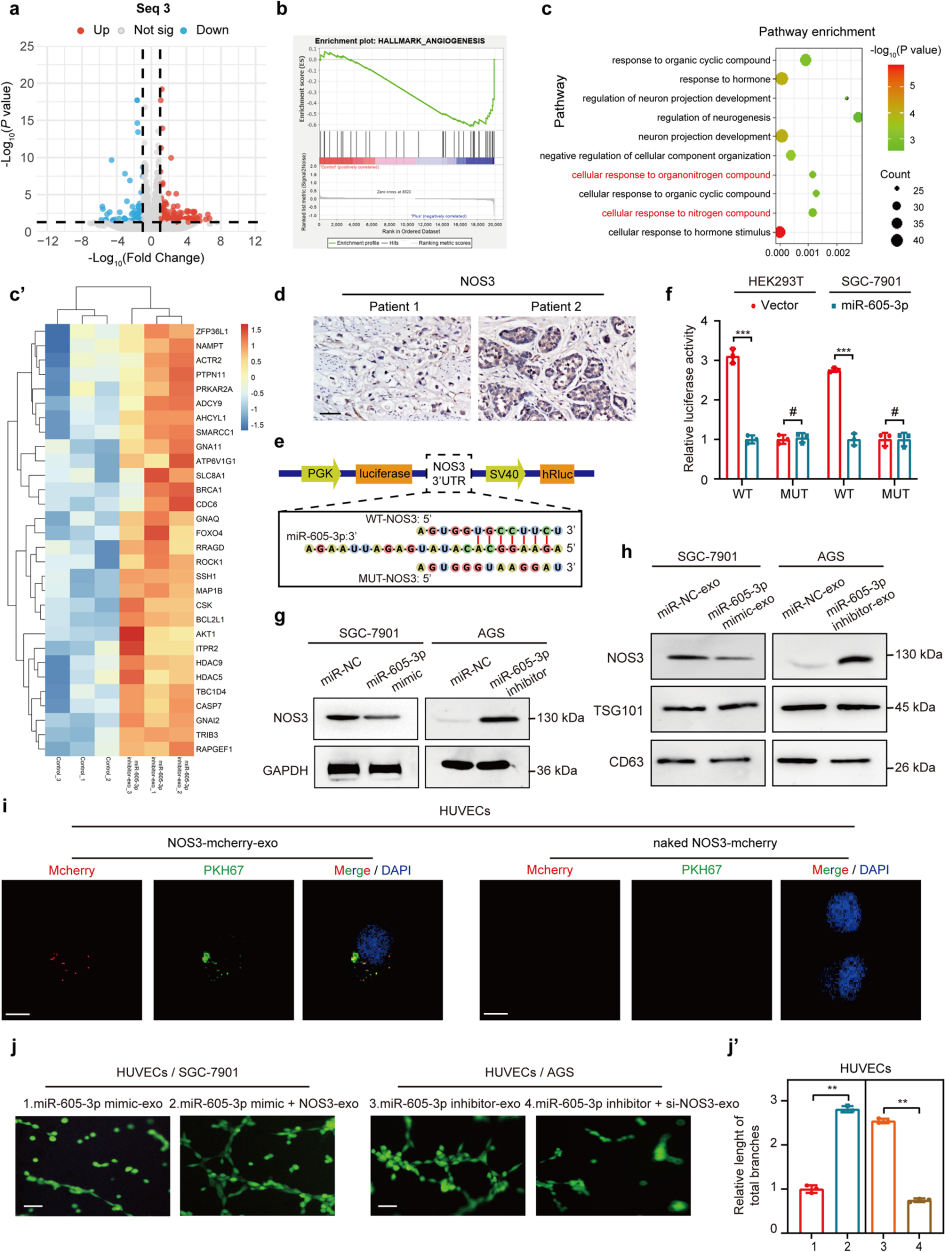
miR-605-3p suppresses angiogenesis by regulating the secretion of exosomal NOS3 from GC cells. **a.** The volcano plot illustrates the results of Seq3 (HUVECs treated with equivalent weight of exosomes obtained from AGS cells and control cells with miR-605-3p inhibitor), with red indicating upregulation, blue indicating downregulation, and gray representing no significant difference. **b.** GSEA generated an ordered list of genes based on genes upregulated in the transcriptome sequencing (HUVECs treated with AGS/miR-605-3p inhibitor-exo vs HUVECs treated with AGS/miR-NC-exo (equivalent weight of exosomes), Seq3) **c.** Bubble plot of GO enrichment pathways based on transcriptome sequencing (Seq3). **c'**. The expression of nitric oxide related genes in seq3. **d**. NOS3 determined by IHC in GC tissues (scale bar, 50 µm). **e.** Predicted target sequence of miR-605-3p in the 3′-UTR of NOS3 and mutants containing altered nucleotides in the seed sequence of miR-605-3p. **f.** Luciferase reporter assay in HEK-293T and SGC-7901 cells transfected with a miR-605-3p mimic and WT or mutated (Mut) NOS3 3’-UTR reporter vector. **g**. WB was performed to evaluate the expression of NOS3 in SGC-7901 and AGS cells with miR-605-3p mimic or inhibitor. **h.** WB was performed to evaluate the expression of NOS3 in 10 µg exosomes from the same group. **i**. Top panels show the fluorescence of HUVECs incubated with PKH67-labelled exosomes derived from SGC-7901 cells for 48 h. HUVECs incubated with naked-NOS3-mCherry were used as a NC (bottom panels) (scale bar, 25 μm). **j**. Tube formation ability of 10 µg exosomes derived from the SGC-7901/miR-605-3p mimic, SGC-7901/miR-605-3p mimic + NOS3, AGS/miR-605-3p inhibitor and AGS/inhibitor + si-NOS3 groups (scale bar, 100 μm). **P < 0.01, ***P < 0.001, ^#^*P* no significance

Subsequently, we further explored whether miR-605-3p regulates angiogenesis through other mechanisms besides inhibiting exosome release. We initially aimed to verify if miR-605-3p was transported directly to HUVECs via exosomes to influence angiogenesis, but qRT-PCR analysis did not detect miR-605-3p in exosomes (Supplementary Fig. 4a). To understand how miR-605-3p mediated exosomes regulate HUVEC angiogenesis, we extracted exosomes of equal quality from miR-605-3p inhibitor AGS cells and control AGS cells, and subsequently performed RNA sequencing on HUVECs treated with these exosomes (Seq3). Transcriptomic analysis (Fig. 5a) and GSEA revealed upregulated genes in the ‘hallmark of angiogenesis’ gene sets (Fig. 5b). GO term analysis showed enrichment of cellular response to organonitrogen and nitrogen compounds (Fig. 5c). NO signature genes were upregulated in HUVECs which treated with exosomes derived from miR-605-3p inhibitor AGS cells (Fig 5c’). The previous literature reports that NOS3 plays a crucial role in regulating NO content in HUVECs [40]. The expression of miR-605-3p also correlated negatively with NOS3 in 108 cases of GC (Fig. 5d, Supplementary Fig. 4b). Thus, we focused on NOS3, and a binding site of miR-605-3p on the NOS3 3′-UTR was predicted by the miRWalk database (Fig. 5e). Luciferase reporter assays confirmed the interaction (Fig. 5f). WB assays showed miR-605-3p mimic downregulated NOS3 expression in SGC-7901 cells, while miR-605-3p inhibitor upregulated NOS3 expression in AGS cells (Fig. 5g). WB assays revealed that, under the condition of equal exosome quality, NOS3 downregulation in exosomes from SGC-7901/miR-605-3p mimic cells and NOS3 upregulation in exosomes from AGS/miR-605-3p inhibitor cells (Fig. 5h, Supplementary Fig. 4c). VAMP3 and NOS3 were investigated independently, and knockdown or overexpression of VAMP3 did not affect NOS3 expression in gastric cancer cells or in exosomes (Supplementary Fig. 4d-f).

To analyze if NOS3 in GC-derived exosomes affects HUVEC function, HUVECs were incubated with mCherry-labeled NOS3-expressing exosomes. Colocalization of mCherry and PKH67 was observed in HUVECs (Fig. 5i). Tube formation assays showed enhanced tube formation ability in HUVECs treated with exosomes of equal quality from miR-605-3p mimic/NOS3 cells compared to those from miR-605-3p mimic cells. Conversely, HUVECs treated with exosomes of equal quality from miR-605-3p inhibitor/si-NOS3 cells exhibited weaker tube formation ability than those from miR-605-3p inhibitor cells (Fig. 5j). Investigation of NOS3-enriched exosomes’ role in NO production in HUVECs revealed reduced NO production in HUVECs treated with exosomes of equal quality from SGC-7901/miR-605-3p cells compared to SGC-7901/miR-NC cells. Exosomes of equal quality from AGS/miR-605-3p inhibitor cells stimulated more NO production. However, the effects of miR-605-3p inhibitor or overexpression in GC cells on NO production in HUVECs were nullified by NOS3 overexpression or knockdown in GC cells, respectively (Supplementary Fig. 4g and 4 h). In summary, NOS3 is present in GC-secreted exosomes and is a direct target of miR-605-3p, contributing to miR-605-3p-mediated effects of GC cell-secreted exosomes on HUVECs.

### Mir-605-3p-mediated release of exosomes promotes PMN formation

**Fig. 6:**
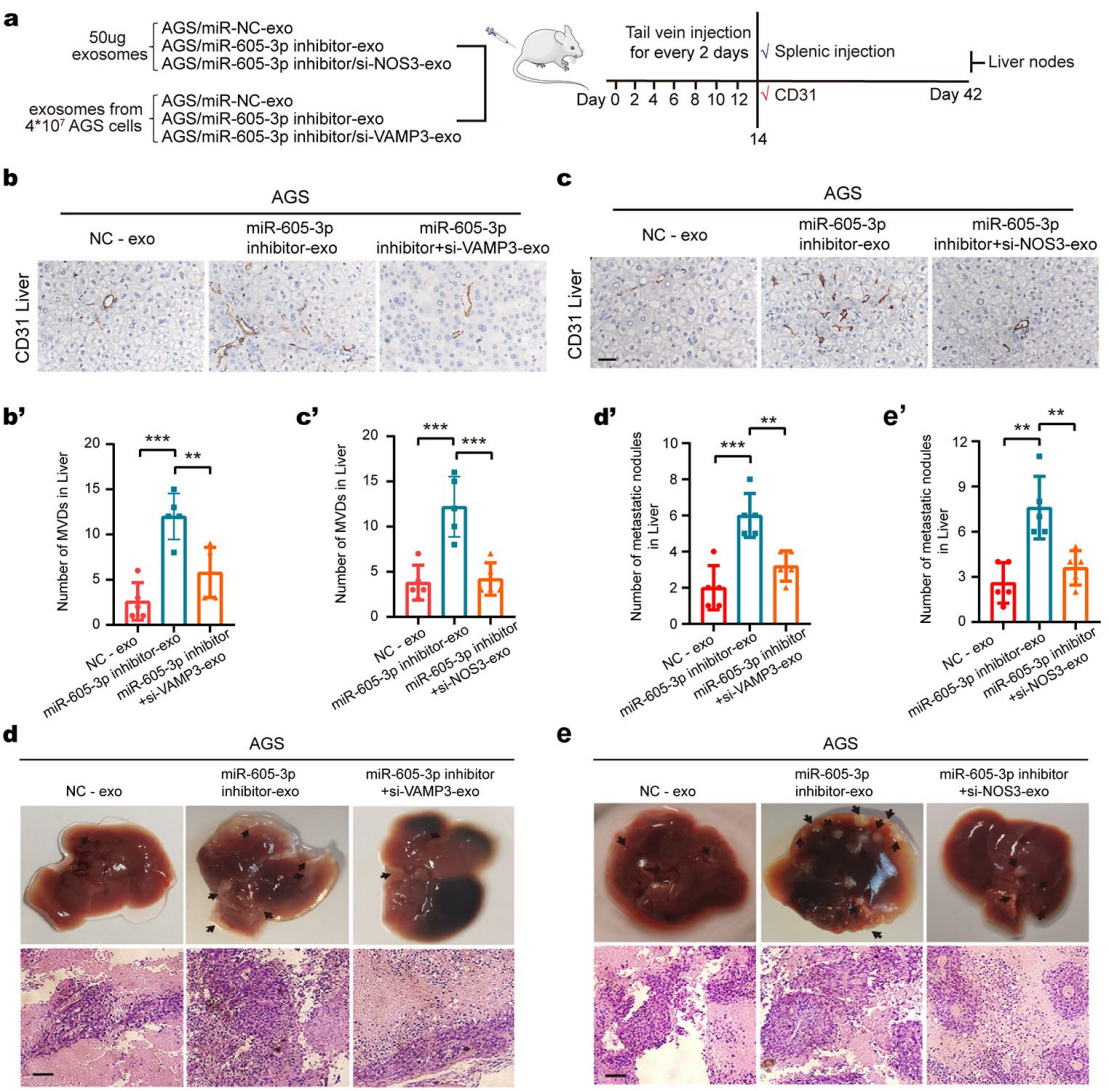
miR-605-3p-mediated release of exosomal NOS3 promotes PMN formation. **a.** Schematic of the experimental design of the mouse model. **b, c.** Effects of exosomes derived from AGS/miR-NC, AGS/miR-605-3p inhibitor, AGS/miR-605-3p inhibitor + si-VAMP3, AGS/miR-605-3p inhibitor + si-NOS3 cells on angiogenesis in the mouse livers were assessed by IHC. **d, e.** Representative images of nodules from the liver of mice with exosomes derived from AGS/miR-NC, AGS/miR-605-3p inhibitor, AGS/miR-605-3p inhibitor + si- VAMP3, AGS/miR-605-3p inhibitor + si-NOS3 cells injected into the intraspleen (liver metastasis). Nodules are indicated by black arrows (the top panels). H&E staining results of the corresponding liver metastatic nodules (bottom panels) are presented (scale bar, 100 μm). The mean ± SEM are provided (*n* = 5). **P < 0.01, ***P < 0.001

Increased angiogenesis in the PMN is crucial for target organ metastasis [41, 42]. We investigated the role of miR-605-3p-mediated GC-secreted exosomes in regulating angiogenesis and PMN formation using a nude mouse model. Exosomes from AGS/miR-NC, AGS/miR-605-3p inhibitor, AGS/miR-605-3p inhibitor/si-VAMP3, and AGS/miR-605-3p inhibitor/si-NOS3 groups were isolated and administered via tail-vein injection every alternate day for 2 weeks, followed by AGS cell implantation in the spleen of nude mice (Fig. 6a). Exosome uptake by vascular endothelial cells in mice was confirmed (Supplementary Fig. 5).

Exosomes from AGS/miR-605-3p inhibitor cells increased liver angiogenesis and tumor liver metastasis compared to exosomes from AGS/miR-NC cells. However, si-VAMP3 reversed the effect of miR-605-3p inhibitor (Fig. 6b, c). Furthermore, exosomes from AGS/miR-605-3p inhibitor cells promoted mouse liver angiogenesis and tumor liver metastasis more than an equal weight of exosomes from AGS/miR-NC cells. However, si-NOS3 reversed the effect of miR-605-3p inhibitor (Fig. 6d, e). These findings indicate that miR-605-3p-mediated exosome secretion accelerates GC cell metastasis by priming PMN formation in the liver through promoting angiogenesis.

### Exosomal NOS3 in plasma is associated with metastasis in GC patients

**Fig. 7:**
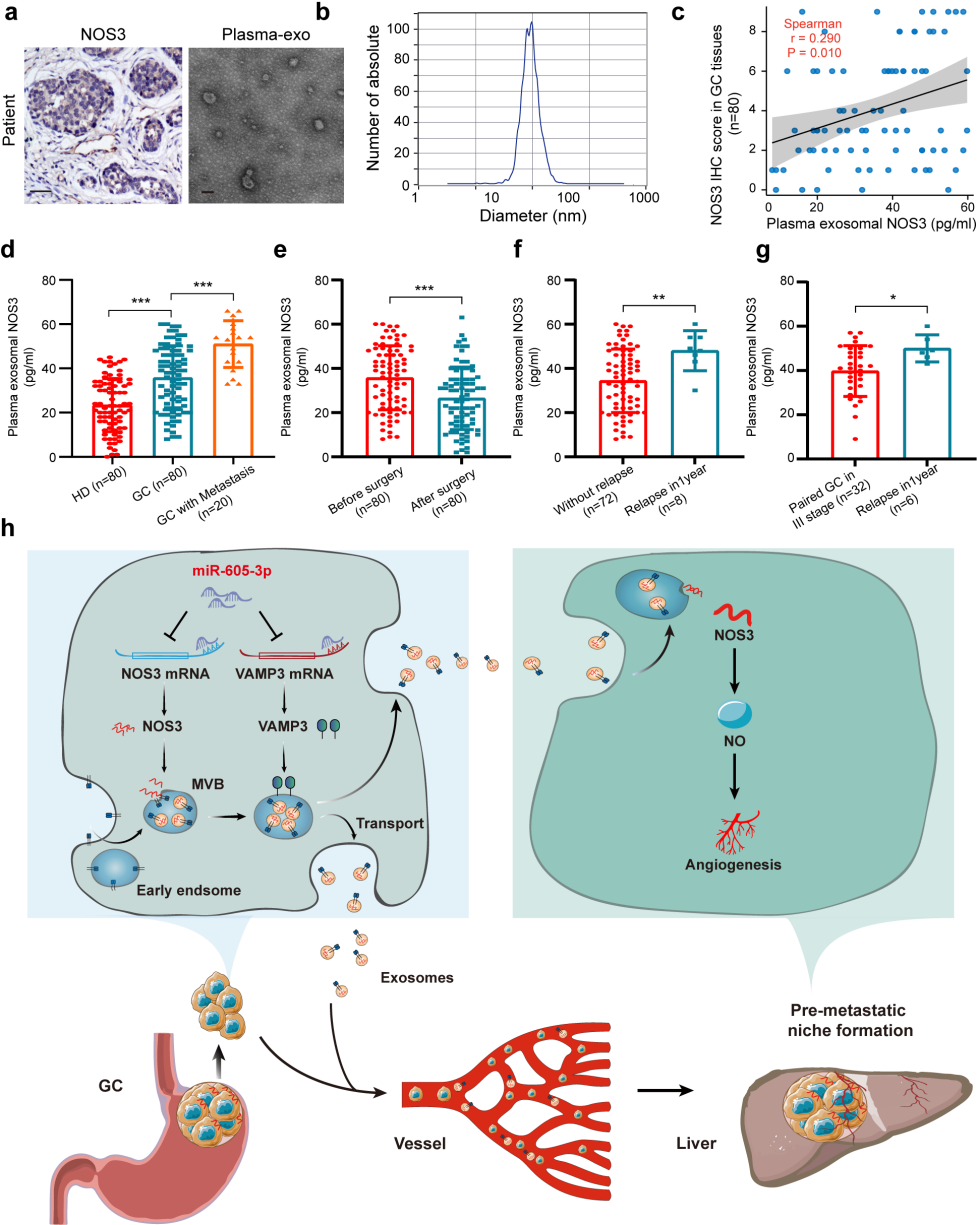
Exosomal NOS3 in plasma is associated with metastasis in GC patients. **a.** TEM of exosomes from the patient plasma (scale bar, 100 nm) and NOS3 expression in tissues as assessed by IHC (scale bar, 50 μm). **b.** Exosome isolation from plasma samples were characterized by NTA. **c.** Pearson’s correlation between exosomal NOS3 in plasma and paired NOS3 expression in tissues (*n* = 80). **d**. ELISA of exosomal NOS3 expression in plasma from 80 GC patients without metastasis, 80 healthy individuals, and 20 GC patients with metastasis. **e.** ELISA of exosomal NOS3 expression in the plasma of 80 GC patients before and after surgery. **f.** ELISA of exosomal NOS3 expression in patients who experienced relapse within one year of diagnosis (*n* = 8) and GC patients who did not experience relapse (*n* = 72). **g**. ELISA of exosomal NOS3 expression in patient plasma who experienced relapse within one year (*n* = 6) and GC patients who did not experience relapse (*n* = 32) and paired based on TNM stage. *P < 0.05, **P < 0.01

To investigate the association of plasma exosomal NOS3 with metastasis in GC, we assessed patients and observed a correlation between exosomal NOS3 levels in plasma and NOS3 expression in paired tissues (Fig. 7a-c). Furthermore, we analyzed plasma exosomal NOS3 levels in GC patients (*n* = 80), which were higher than those in healthy donors (*n* = 80) but significantly lower than in GC patients with metastasis (*n* = 20; Fig. 7d). Moreover, plasma exosomal NOS3 levels were significantly reduced one-week post-surgery compared to pre-surgery levels (Fig. 7e). Additionally, the plasma exosomal NOS3 level was higher in the eight patients who experienced relapse within a year compared to patients without relapse (*n* = 72; Fig. 7f). Notably, considering the TNM stage, exosomal NOS3 was also elevated in relapsed patients (*n* = 6, TNM = 3) compared to stage-matched GC patients (*n* = 32; Fig. 7g). Thus, our clinical data suggest that elevated levels of plasma exosomal NOS3 are associated with GC metastasis.

## Discussion

Angiogenesis plays a pivotal role in establishing PMNs by delivering essential nutrients and oxygen required for tumor proliferation and enabling the entry of circulating tumor cells [43]. Exosomes derived from GC facilitate angiogenesis by transporting diverse miRNAs that modulate gene expression critical to this process [44]. Moreover, Tetraspanin 8, a transmembrane protein found on these GC-derived exosomes, activates endothelial cells through the MAPK signaling pathway [45]. Exosomal YB-1, also from GC, further promotes angiogenesis by elevating levels of angiogenic factors [46]. Our research introduces a novel concept whereby primary GC-derived exosomes modulate organotropic metastasis through both the release mechanisms of the exosomes and their functional cargo. We have found that miR-605-3p specifically targets and downregulates VAMP3, a critical component of the SNARE complex essential for exosome release. This inhibition significantly limits exosomal export from GC cells. Furthermore, miR-605-3p directly suppresses the expression of NOS3, a vital angiogenic factor, reducing its abundance in exosomes and consequently impairing angiogenic capabilities in HUVECs. This reduction in angiogenic potential, stemming from decreased NOS3 levels in exosomes, obstructs the development of a pre-metastatic niche in the liver, thereby effectively inhibiting liver metastasis.

VAMP3 is a critical component of the SNARE protein complex responsible for mediating vesicular trafficking and membrane fusion events. Recently, its role in the biogenesis and release of extracellular vesicles, particularly exosomes, has drawn significant attention. The SNARE-mediated membrane fusion, where VAMP3 acts as a v-SNARE on vesicle membranes, is paramount for ensuring cargo transfer and vesicle release. As a target gene of miR-124, reduced VAMP3 expression stimulates neuroblastoma cell proliferation and suppresses apoptosis [47]. A concomitant decrease in VAMP3, regulated by increased miR-124 levels, leads to reduced inflammatory cytokine release associated with microglial activation post-surgery [48]. Furthermore, impeding VAMP3 recycling with WDFY2 diminishes the secretion of matrix metalloproteinases by cancer cells, necessary for remodeling the extracellular matrix. This process enables cancer cells to overcome tissue barriers and form metastases [49]. These findings highlight the multifactorial roles of VAMP3 in various pathological processes, especially in cancer biology, demonstrating its significance in roles independent of exosomes. In liver cancer and pancreatic cancer, the complex of VAMP3 plays a crucial role in mediating the membrane fusion process prior to exosome secretion [11, 13]. However, the role of VAMP3 as an exosome secretion regulator in GC, and its underlying mechanisms, remain unclear. In our current study, VAMP3 acts as a target gene of miR-605-3p, leading to an increase MVBs and ILVs in GC cells. This effect is observed without significant changes in the morphology or the function of early endosomes, lysosomes, and autophagosomes.

NOS3 is a unique NO synthase in vascular endothelial cells, and NOS3 polymorphisms are related to the susceptibility of individuals to cardiovascular diseases [50]. Its main function is to form dimers under the action of calcium ions and undergo phosphorylation modification to decompose arginine and proline and generate NO [51, 52]. As a second messenger, NO continues to transmit signals through cGMP, thereby regulating vascular tension; activating endothelial progenitor cells; promoting the proliferation and migration of endothelial cells, the construction of a vascular network and the formation of vascular cavities; and enhancing local regeneration through angiogenesis [53]. The expression of NOS3 in vascular endothelial cells is regulated in many ways. During oxidative stress or ischaemia–reperfusion, the hypoxic state of the tissue microenvironment can not only improve the stabilization of NOS3 mRNA by weakening the function of target miRNAs but also produce cytokines or induce the expression of NOS3 in endothelial cells [54], thereby improving the NO production capacity. During the occurrence of preeclampsia, plasma exosomal miR-155 derived from the placenta can be internalized by endothelial cells, thereby inhibiting NOS3 expression in the vascular endothelium, reducing NO production, and consequently affecting the progression of preeclampsia [55]. Our research findings indicate that, in addition to the previously identified regulatory mechanisms of NOS3 within endothelial cells themselves, we have discovered that endothelial cells can uptake NOS3 from tumor-secreted exosomes. This process facilitates aginogenesis in the PMNs. This mechanism highlights how tumors ingeniously exploit body systems, such as the vascular system, to advance their own growth and spread. Furthermore, when we analyzed plasma exosomal NOS3 levels, we found that these were higher in the patients who experienced a relapse within a year compared to those without relapse. These results suggest that high plasma exosomal NOS3 levels are associated with increased angiogenesis, leading to GC relapse. On one hand, we hypothesize that exosomal NOS3 could serve as an indicator of GC relapse. On the other hand, patients with high exosomal NOS3 levels may be more vulnerable to therapies with vascular inhibitors. However, this hypothesis requires further investigation to confirm.

## Conclusion

we showed that low expression of miR-605-3p is an independent predictor of poor prognosis in GC. Reduced miR-605-3p expression leads to upregulated expression of VAMP3 and NOS3 in GC cells. Increased VAMP3 expression promotes exosome secretion, and NOS3 released from these exosomes can be internalized by HUVECs in metastatic organs, promoting local angiogenesis and increasing GC cell metastasis. Additionally, the plasma concentration of exosomal NOS3 can serve as an indicator of GC metastasis. These findings enhance our understanding of the molecular mechanisms underlying GC metastasis and provide a basis for the prevention and treatment of GC.

### Electronic supplementary material

Below is the link to the electronic supplementary material.

Supplementary Material 1

Supplementary Material 2

Supplementary Material 3

Supplementary Material 4

Supplementary Material 5

Supplementary Material 6

## Data Availability

The data that support the findings of this study are available on request from the corresponding author.

## Abbreviations

GC: Gastric cancer
PMN: Pre-metastatic niche
NOS3: Nitric oxide synthase 3
VAMP3: Vesicle-associated membrane protein 3
EVs: Extracellular vesicles
MVBs: Multivesicular bodies
ISV: Intersegmental vessel
VEGF: Vascular endothelial growth factor
ILVs: Intraluminal vesicles
OS: Overall survival
DFS: Disease-free survival
Rab: Ras-related proteins in brain
SNARE: Soluble NSF-attachment protein receptor
ASC: Adipose-derived stem cell
TMOD3: Tropomodulin3
MMP: Matrix metalloproteinases
