# Supplementary figures and images for: mir-605-3p prevents liver premetastatic niche formation by inhibiting angiogenesis via decreasing exosomal nos3 release in gastric cancer
Source: Cancer Cell Int. 2024 May 27;24:184. doi: 10.1186/s12935-024-03359-5 (PMC11131241; doi:10.1186/s12935-024-03359-5)

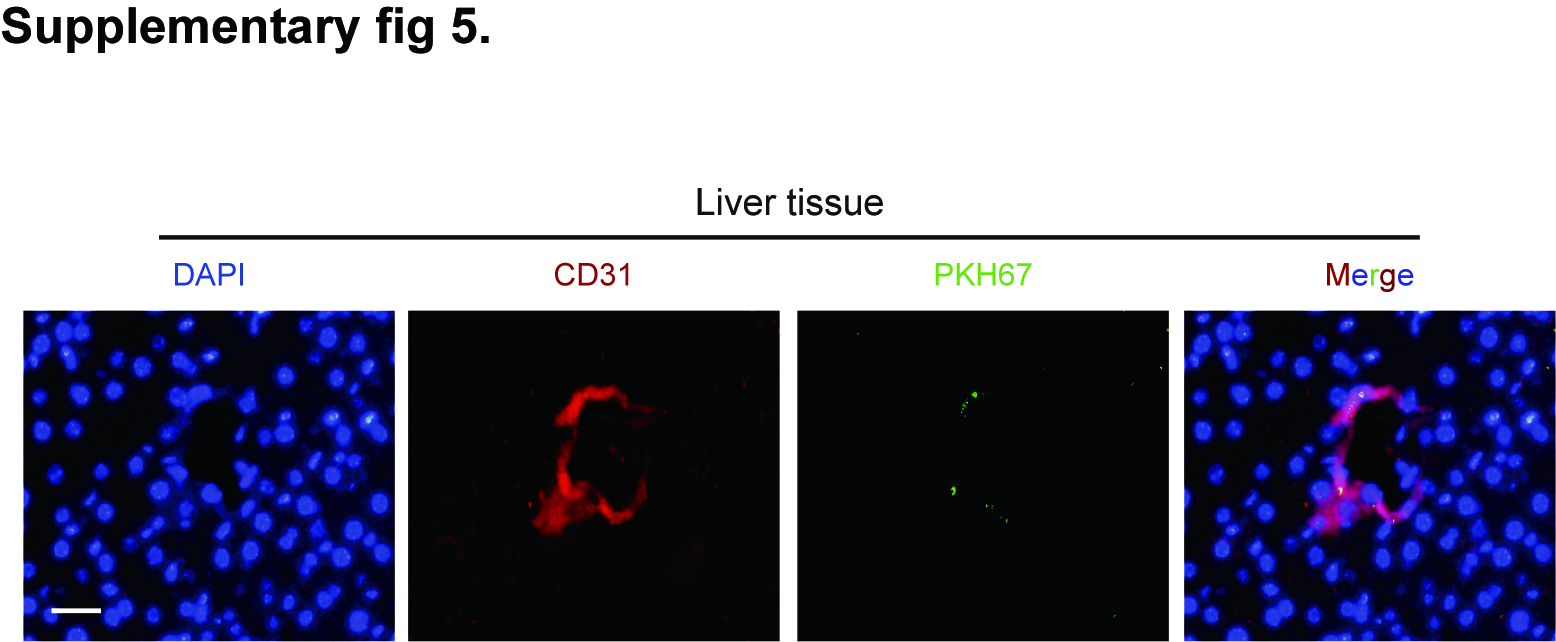

Supplement: Supplementary file 1 — Supplementary Material 1 [file 12935_2024_3359_MOESM1_ESM.tif]

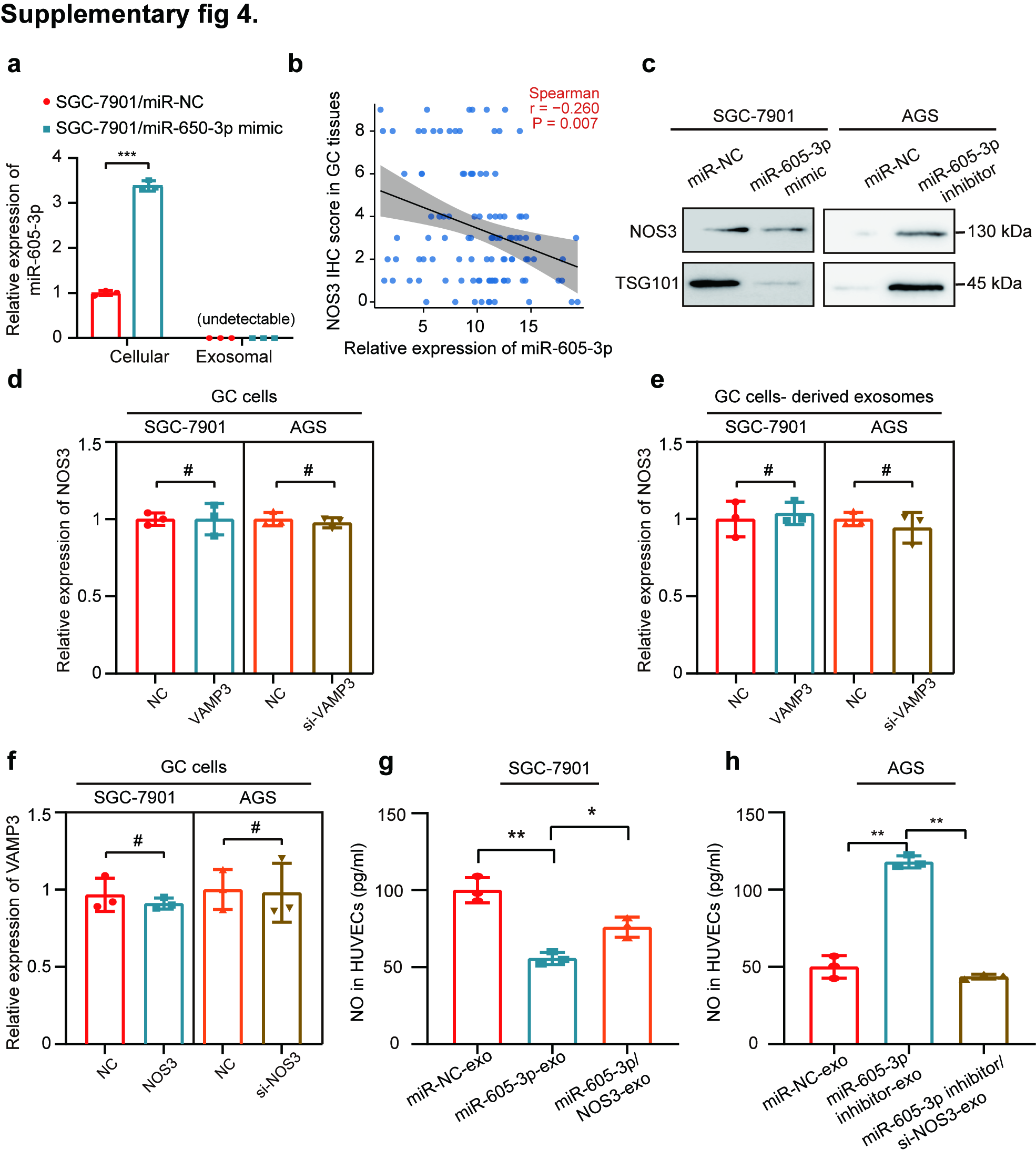

Supplement: Supplementary file 2 — Supplementary Material 2 [file 12935_2024_3359_MOESM2_ESM.tif]

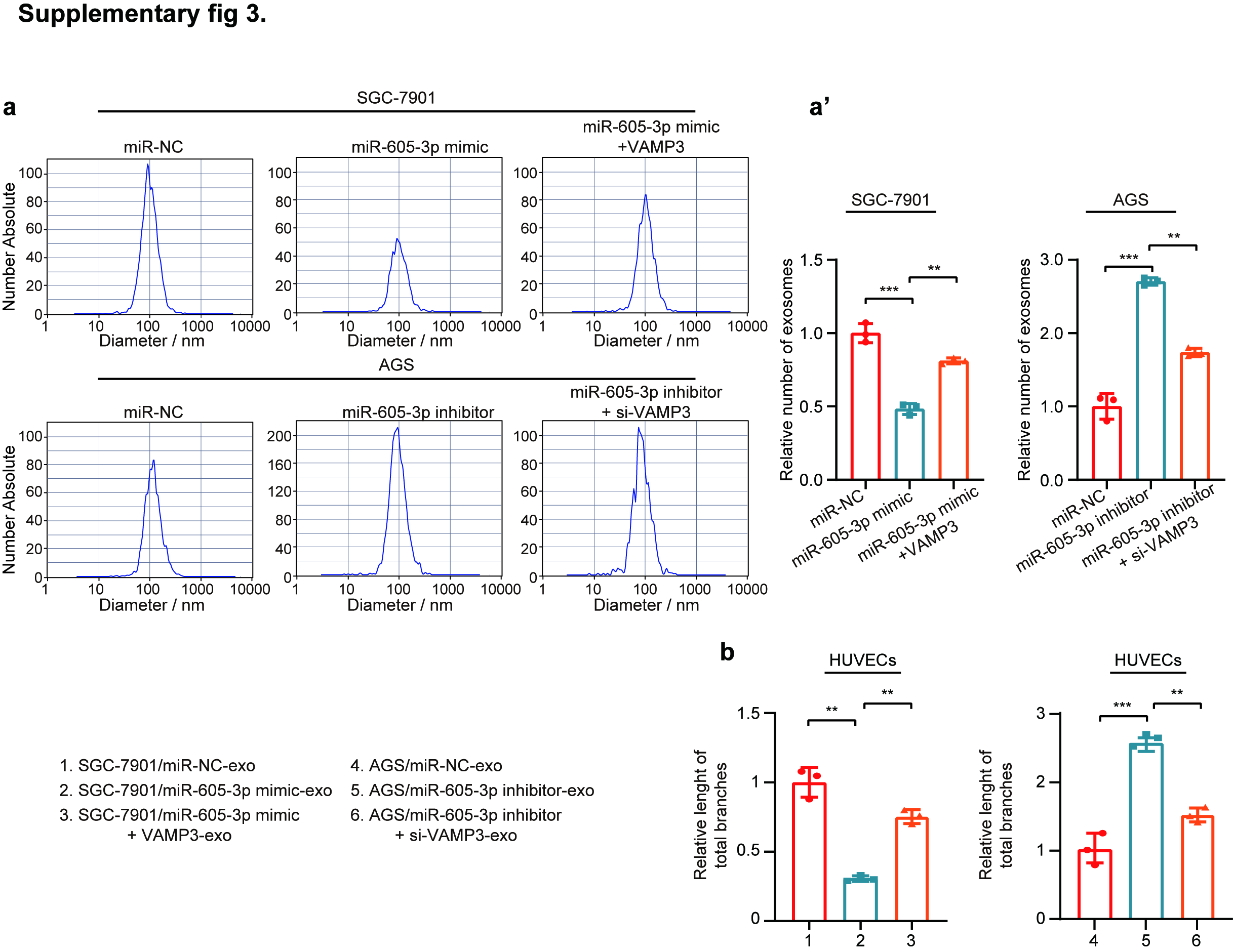

Supplement: Supplementary file 3 — Supplementary Material 3 [file 12935_2024_3359_MOESM3_ESM.tif]

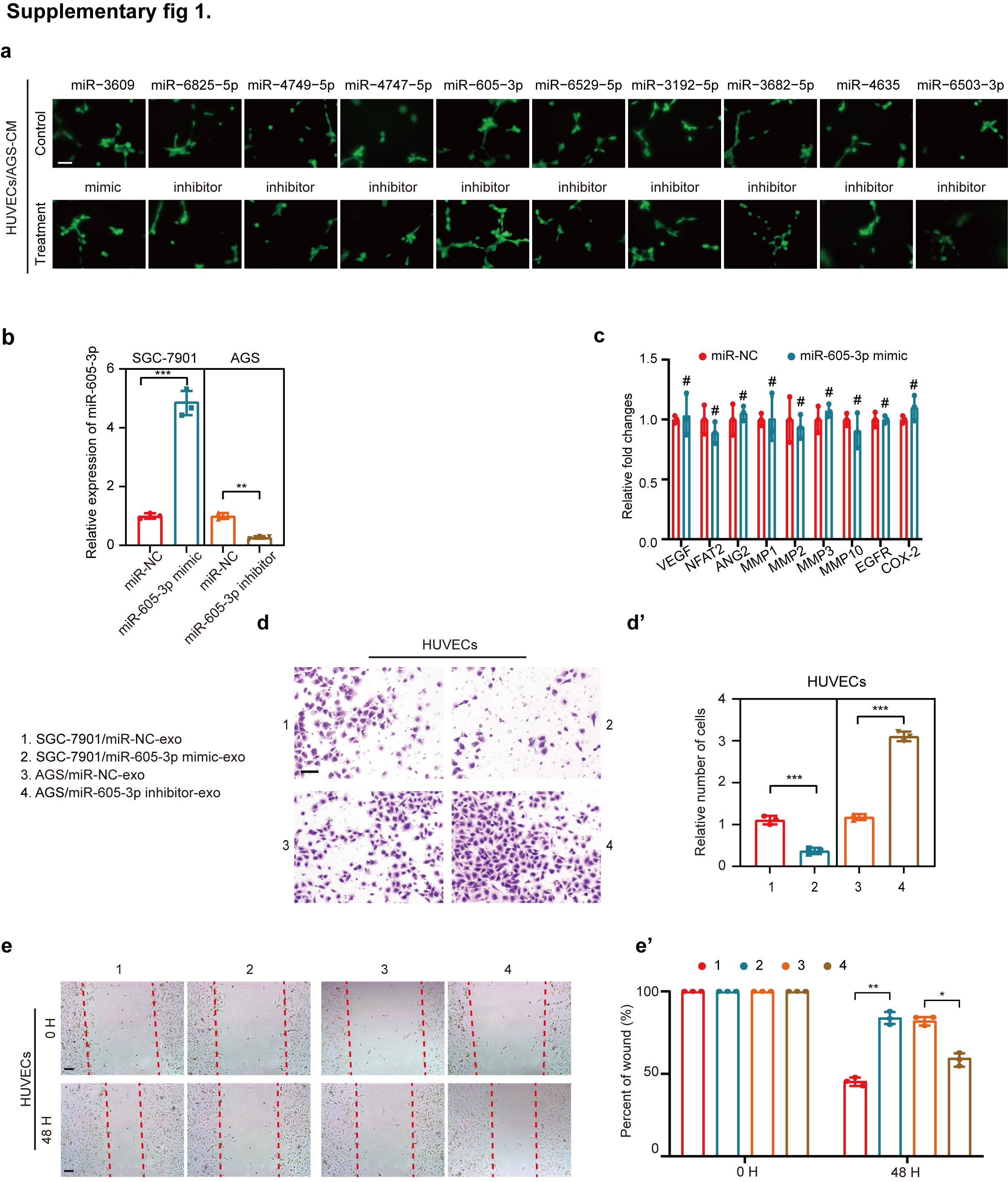

Supplement: Supplementary file 4 — Supplementary Material 4 [file 12935_2024_3359_MOESM4_ESM.tif]

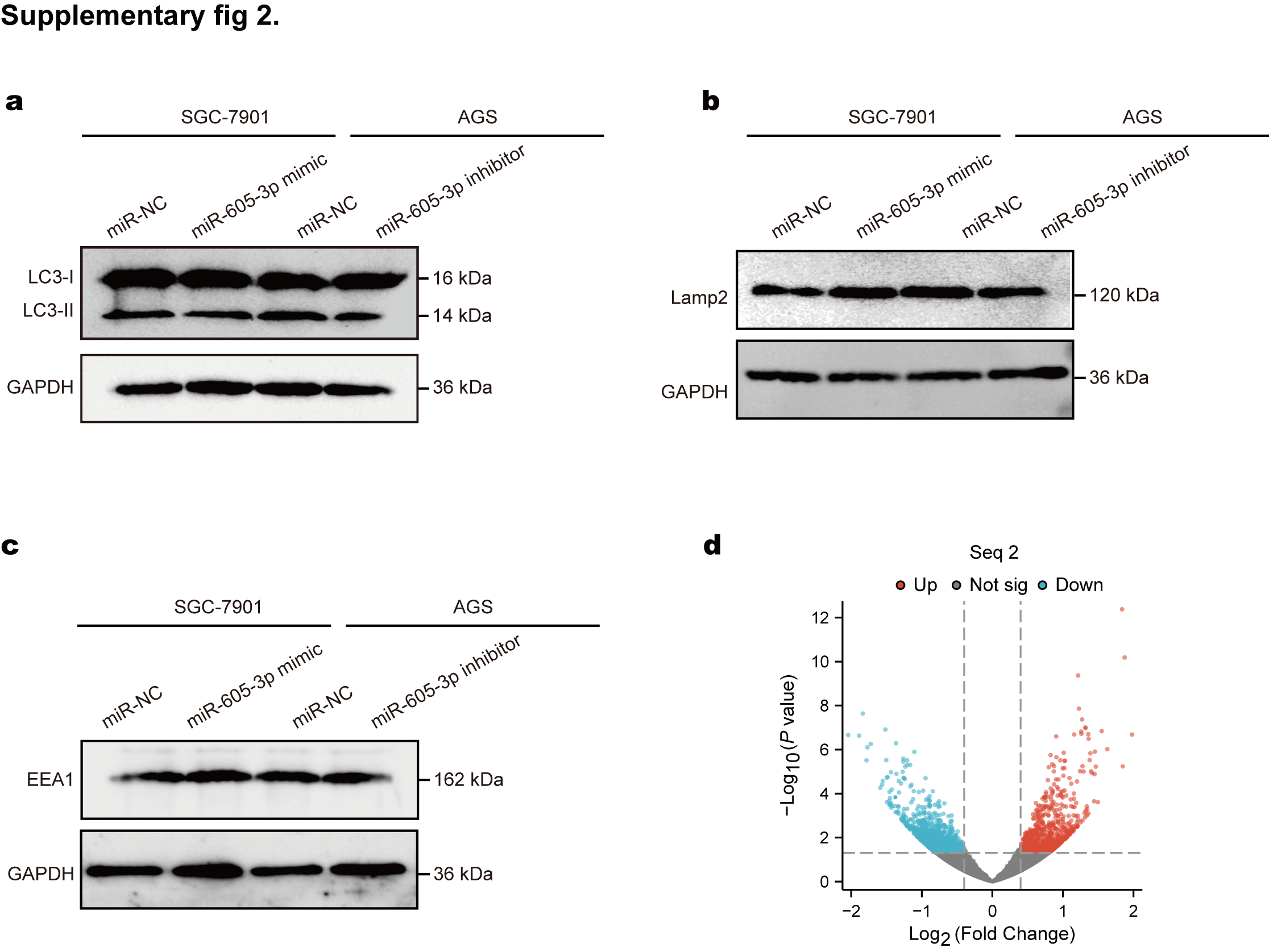

Supplement: Supplementary file 6 — Supplementary Material 6 [file 12935_2024_3359_MOESM6_ESM.tif]
