# Supplementary material for: mir-605-3p prevents liver premetastatic niche formation by inhibiting angiogenesis via decreasing exosomal nos3 release in gastric cancer
Source: Cancer Cell Int. 2024 May 27;24:184. doi: 10.1186/s12935-024-03359-5 (PMC11131241; doi:10.1186/s12935-024-03359-5)
Supplement: Supplementary file 5 — Supplementary Material 5 [file 12935_2024_3359_MOESM5_ESM.docx]

**Supplementary Figure Legends**

**Supplementary Fig 1. miR-605-3p suppresses HUVECs metastasis and tube formation.**

**a.** Effects of conditioned medium (CM) derived from AGS cells treated with miR-NC, miR-3609 mimic, miR-6825-5p inhibitor, miR-4749-5p inhibitor, miR-4747-5p inhibitor, miR-605-3p inhibitor, miR-6529-5p inhibitor, miR-3192-5p inhibitor, miR-3682-5p inhibitor, miR-4635 inhibitor, miR-6503-3p inhibitor on the tube formation ability of HUVECs (scale bar, 100 µm). **b.** miR-605-3p levels in SGC-7901/miR-NC, SGC-7901/miR-605-3p mimic, AGS/miR-NC or AGS/ miR-605-3p inhibitor as detected by qRT-PCR. **c**. Expression of angiogenesis-associated factors as detected by qRT-PCR. **d.** Transwell assay showing the migration of HUVECs treated with exosomes derived from the same number of SGC-7901/miR-NC, SGC-7901/miR-605-3p, AGS/miR-NC, and AGS/miR-605-3p inhibitor cells (scale bar, 100 μm). **e**. Migration of HUVECs treated with the same exosomes as assessed by the wound healing assay (scale bar, 100 μm). #P>0.05, *P<0.05, **P<0.01, ***P<0.001.

**Supplementary Fig 2. WB detection of the levels of markers of typical intracellular vesicles.**

**a-c.** WB was used to detect the expression levels of Lamp2, EEA1 and LC3B in the same number of cells. d. The volcano plot illustrates the results of Seq2, with red indicating upregulation, blue indicating downregulation, and gray representing no significant difference.

**Supplementary Fig 3. WB detection of the protein levels of Rab and SNARE family members.**

**a.** Characterization and quantitation of vesicles derived from same number of SGC-7901/miR-NC, SGC-7901/miR-605-3p mimic, SGC-7901/miR-605-3p mimic + VAMP3, AGS/miR-NC, AGS/miR-605-3p inhibitor, and AGS/miR-605-3p inhibitor + si-VAMP3 cells as assessed by NTA. **b**. Quantitation of the tube formation ability of HUVECs treated with exosomes derived from the same number of GC cells (scale bar, 100 µm). **P<0.01, ***P<0.001.

**Supplementary Fig 4. miR-605-3p suppresses angiogenesis by targeting NOS3 and regulating exosomal NOS3 derived from GC cells**

**a**. qRT-PCR was used to detect the expression of miR-605-3p in GC cells and exosomes. **b**. Expression of miR-605-3p correlated negatively with that of NOS3 in 108 cases of GC. **c.** WB detection of the protein levels of NOS3 and TSG101 in exosomes derived from the same number of SGC-7901/miR-NC, SGC-7901/miR-605-3p mimic, AGS/miR-NC, and AGS/miR-605-3p inhibitor cells. **d.** qRT-PCR was used to detect the expression level of NOS3 in SCG-7901/VAMP3 and AGS/si-VAMP3. **e.** qRT-PCR was used to detect the expression level of NOS3 in exosomes of the same weight from SCG-7901/VAMP3 and AGS/si-VAMP3. **f.** qRT-PCR was used to detect the expression level of VAMP3 in SCG-7901/NOS3 and AGS/si-NOS3. **g, k.** NO expression in exosomes derived from HUVECs of different treatment groups as assessed by ELISA. **P<0.01, ***P<0.001.

**Supplementary Fig 5. PKH67-labeled exosomes were ingested by vascular endothelial cells in the liver of nude mice.**

Fluorescence image of the liver of a nude mouse injected with PKH67-labeled exosomes through the tail vein (scale bar, 100 μm).

**Supplementary Tables：**

***Table S1. Target sequences***

| **Name** | Target Seq |
| --- | --- |
| **si-NOS3** | CAGCAGUGGAAAUCAACGUTT  ACGUUGAUUUCCACUGCUGTT |
| **si-VAMP3** | CACUGUAAUCACCUAAAUAAATT |
| **miR-605-3p inhibitor** | UCUAAAUCUCAUAGUGCCUUCU |
| **miR-605-3p mimic** | AGAAGGCACUAUGAGAUUUAGA  UAAAUCUCAUAGUGCCUUCUUU |

***Table S2. Sequences of primers used for amplification of target genes***

| Gene primer nucleotide sequence |
| --- |
| miR-605-3p Forward: 5′- AACGAGACGACGACAGAC -3′  Reverse: 5′- AGAAGGCACTATGAGATTTAGA -3′  VEGF Forward: 5′- ATGAACTTTCTGCTGTCTTGGGT -3′  Reverse: 5′- TGGCCTTGGTGAGGTTTGATCC -3′  NFAT2 Forward: 5′- -GCTATGCATCCTCCAACGTC -3′  Reverse: 5′- AGTTFFACTCGTAGGAGGAG -3′  ANG2 Forward: 5′- GGCAGCGTTGATTTTCAGAGGACT -3′  Reverse: 5′- TTTAATGCCGTTGAACTTATTTGT -3′  MMP1 Forward: 5′- AGAGAGCAGCTTCAGTGACA -3′  Reverse: 5′- -CTTGAGCTGCTTTTCCTCCG -3′  MMP2 Forward: 5′- TTGACGGTAAGGACGGACTC -3′  Reverse: 5′- ACTTGCAGTACTCCCCATCG -3′  MMP3 Forward: 5′- ATTCCATGGAGCCAGGCTTTC -3′  Reverse: 5′- CATTTGGGTCAAACTCCAACTGT -3′  MMP10 Forward: 5′- GATCTTGCTCAGCAATACCTAG -3′  Reverse: 5′- TCACAATCCTGTAGGAGATGTG -3′  EGFR Forward: 5′- TGGTCAAGTGCTGGATGATAGA -3′  Reverse: 5′- ACGGTAGAAGTTGGAGTCTGTA-3 -3′  COX2 Forward: 5′- TGCAGTGAGCGTCAGGAG -3′  Reverse: 5′- CAAGGATTTGCTGTATGGCTGAG -3′  VAMP3 Forward: 5′- ATGTCTACAGGTCCAACTGC -3′  Reverse: 5′- TCATGAAGAGACAACCCACA -3′  GDPDH Forward: 5′- GAGTCAACGGATTTGGTCGT -3′  Reverse: 5′- TGGGTGGAATCATATTGGAA -3′  U6 Forward: 5′- ATTGGAACGATACAGAGAAGATT -3′  Reverse: 5′- GGAACGCTTCACGAATTT G -3′ |
